# Congenital Zika Virus Infection in Immunocompetent Mice Causes Postnatal Growth Impediment and Neurobehavioral Deficits

**DOI:** 10.3389/fmicb.2018.02028

**Published:** 2018-08-29

**Authors:** Amber M. Paul, Dhiraj Acharya, Biswas Neupane, E. Ashely Thompson, Gabriel Gonzalez-Fernandez, Katherine M. Copeland, Me’Lanae Garrett, Haibei Liu, Mariper E. Lopez, Matthew de Cruz, Alex Flynt, Jun Liao, Yan-Lin Guo, Federico Gonzalez-Fernandez, Parminder J. S. Vig, Fengwei Bai

**Affiliations:** ^1^Department of Biological Sciences, University of Southern Mississippi, Hattiesburg, MS, United States; ^2^Department of Bioengineering, University of Texas, Arlington, TX, United States; ^3^Hattiesburg Clinic, Hattiesburg, MS, United States; ^4^Department of Neurology, University of Mississippi Medical Center, Jackson, MS, United States; ^5^Medical Research Service, G.V. (Sonny) Montgomery Veterans Affairs Medical Center, Jackson, MS, United States; ^6^Department of Ophthalmology and Pathology, University of Mississippi Medical Center, Jackson, MS, United States; ^7^Pathrd, Inc., Jackson, MS, United States

**Keywords:** Zika, postnatal development, wild-type mice, neuron, behavior

## Abstract

A small percentage of babies born to Zika virus (ZIKV)-infected mothers manifest severe defects at birth, including microcephaly. Among those who appeared healthy at birth, there are increasing reports of postnatal growth or developmental defects. However, the impact of congenital ZIKV infection in postnatal development is poorly understood. Here, we report that a mild congenital ZIKV-infection in pups born to immunocompetent pregnant mice did not display apparent defects at birth, but manifested postnatal growth impediments and neurobehavioral deficits, which include reduced locomotor and cognitive deficits that persisted into adulthood. We found that the brains of these pups were smaller, had a thinner cortical layer 1, displayed increased astrogliosis, decreased expression of microcephaly- and neuron development- related genes, and increased pathology as compared to mock-infected controls. In summary, our results showed that even a mild congenital ZIKV infection in immunocompetent mice could lead to postnatal deficits, providing definitive experimental evidence for a necessity to closely monitor postnatal growth and development of presumably healthy human infants, whose mothers were exposed to ZIKV infection during pregnancy.

## Introduction

Despite being discovered about 70 years ago, ZIKV had not gained much public health attention until its massive outbreak in Brazil in 2015, whereby it has been suggested as a causative agent of microcephaly and other congenital birth defects in human newborns whose mothers were exposed to ZIKV during their first trimester of pregnancy (Campos et al., 2015). In addition, ZIKV infection can cause *Guillain*-*Barré* syndrome and meningoencephalitis in adults (Oehler et al., 2014; Carteaux et al., 2016). ZIKV is primarily transmitted to humans by the *Aedes* species of mosquitos (Hayes, 2009), but can also be acquired through other routes, such as sexual intercourse (D’Ortenzio et al., 2016; Tabata et al., 2016). Currently, ZIKV infection is causing significant public health concerns, however, its pathogenesis is incompletely understood, and there is no approved therapeutic or vaccine available.

Studies have documented that ZIKV primarily targets both human and mouse neural progenitor cells, resulting in cell death and disruption of neurogenesis, including neuronal proliferation, differentiation, and migration (Dang et al., 2016; Garcez et al., 2016; Li C. et al., 2016; Nowakowski et al., 2016; Tang et al., 2016). For instance, direct inoculation of ZIKV into the lateral ventricle of mouse fetuses (C57 or ICR strain), or into the amniotic fluid of the uterus (C57BL/6) (Cui et al., 2017), or intraperitoneal (i.p.) inoculation of pregnant mice (C57 or C57BL/6) at embryonic days 13.5–15.5 can result in disruption of fetal neural progenitor cells and cortical development (Li C. et al., 2016; Shao et al., 2016; Wu et al., 2016). Immunocompetent mice are less susceptible to ZIKV-mediated complications because ZIKV does not antagonize the mouse type I interferon response like it does in humans (Grant et al., 2016; Lazear et al., 2016; Mounce et al., 2016; Rossi et al., 2016). It has been shown, the non-structural protein NS5 of ZIKV selectively degrades the IFN-regulated transcriptional activator STAT2 in humans, but not in mice (Grant et al., 2016). Therefore, recent studies used various immunodeficient or immune-manipulated mouse strains to study the impact of congenital ZIKV infection on fetal development. For instance, ZIKV infection in pregnant SJL mice (T and B cell dysfunction) or mice with partial interferon function deficiency, resulted in fetal demise, intrauterine growth restriction, and signs of microcephaly in newborns, while no apparent anomalies were observed in pups from ZIKV-infected (i.p., intravenously, or subcutaneously) immunocompetent (C57BL/6) dams at birth (Cugola et al., 2016; Miner et al., 2016). Yet, data from these studies may not recapitulate most human infections due to the immune compromised state of the mice. Recently, post-natal behavioral abnormalities were observed in (C57BL/6) mice that were inoculated directly within the amniotic fluid of the uterus, resulting in ocular deficits that contributed to behavioral deficits, however viral RNA was detected in fetal brains at birth and at day 8 post-birth (Cui et al., 2017), indicating viral infection lasted into postnatal development in this model. In contrast, the majority of human infants born to mothers who were exposed to ZIKV during their first trimester of pregnancy were free of viral RNA and appeared healthy at birth, as the chance of developing congenital microcephaly, is between 13–15 percent (Cauchemez et al., 2016; Johansson et al., 2016; Reynolds et al., 2017). Considering the severe effects of ZIKV infection on neural progenitor cells, it is possible that even a transient congenital ZIKV infection during embryonic development could result in long-term defects during postnatal developmental, even if no prominent symptoms are observed at birth. This also holds true for other congenital viral infections, such as cytomegalovirus (CMV) and rubella, which can both cause postnatal manifestations in infants, such as seizures, hearing loss and neurodevelopmental delays, with no obvious abnormalities noticed at birth (Sever et al., 1985; Fowler et al., 1992). Furthermore, the contribution and/or degree of maternal and fetal inflammation triggering neurodegeneration *in utero* must also be considered in order to better understand the mechanisms involved neuropathological progression post birth (Bilbo and Schwarz, 2009; Felix et al., 2017; Vermillion et al., 2017; Hirsch et al., 2018). Most importantly, recent evidence has surfaced that some normal appearing neonates born to ZIKV-infected pregnant women have developed postnatal microcephaly symptoms including brain neuroimaging abnormalities, head growth restrictions, and behavioral deficits months after birth (Licon, 2016; van der Linden et al., 2016). In addition, the complex involvement of co-morbid neurodevelopmental sequelae of infants that were exposed to ZIKV *in utero* has also been described in infants months to years after birth; for example, motor abnormalities, such as dyskinesia, hypertonia, and spasticity, have been observed together with seizures (Pessoa et al., 2018). In line with this, intra-amniotic infection of immunocompetent murine dams identified co-morbid motor deficits, such as rota-rod latency and gait/stride disturbances in pups, which may have been attributed to vision disparities (Cui et al., 2017). Therefore, there is an urgent need to develop an animal model that mimics human infection, to study the long-term developmental consequences of congenital ZIKV infection. Considering that ZIKV infection in immunocompetent mice is transient and self-limiting, we used this mouse model to assess potential ZIKV-associated postnatal developmental sequelae. Our results showed that newborn pups from ZIKV infected immunocompetent dams were free of ZIKV RNA and had similar head size and body weight with mock-infected control pups, yet manifested postnatal growth impediments and neurobehavioral deficits that persisted into adulthood. Thus, our mouse model can be used to mimic and evaluate the potential outcomes of a mild, transient prenatal ZIKV infection in human babies.

## Results and Discussion

**FIGURE 1:**
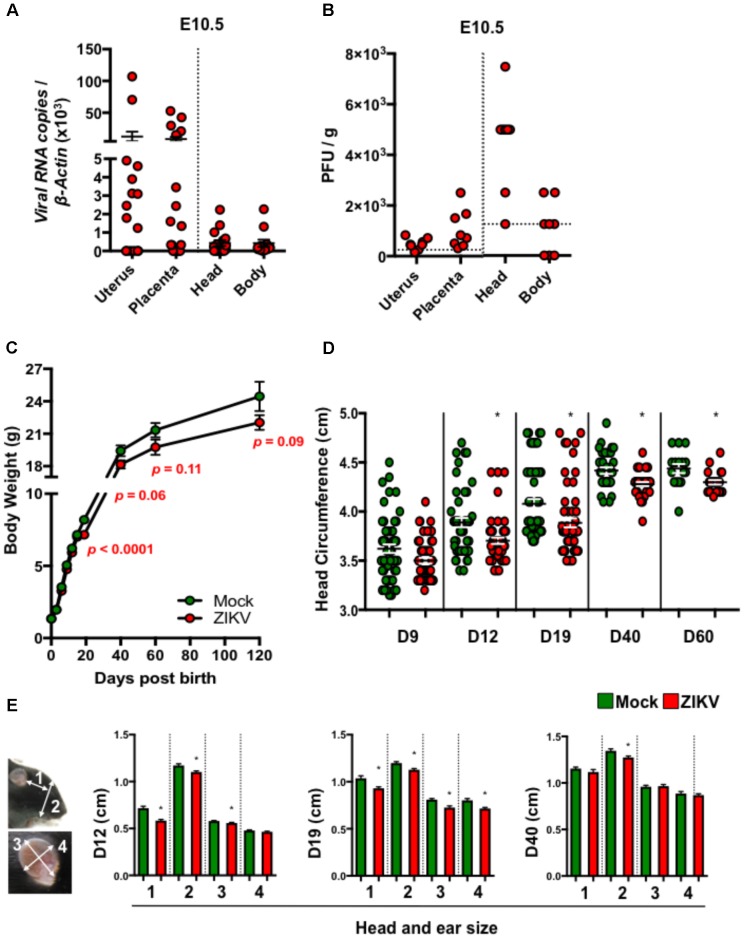
ZIKV-pups exhibit postnatal growth impediment. Dams were infected with ZIKV (10^4^ PFU, i.p.) or mock infected on E8.5. On D2 p.i. (E10.5), ZIKV-*E* RNA **(A)** or infectious virions **(B)** in maternal uterus, placenta, fetal head and fetal body (*n* = 4–6 dams) were measured by qPCR or plaque assay (horizontal dash lines denote detection limit). **(C)** Pup body weight in grams (g) (*n* = 11–65). **(D)** Head circumference of pups (*n* = 11–58). **(E)** Pup head and ear size measurements: “1”, length between outer corner of eye to base of ear; “2”, length from base of chin to top of head; “3”, length of ear from base to top (midline); and “4”, width of ear, *n* = 20–59 at D12, D19, and D40 p.b. All head and body measurements were blinded from the investigator handling the mice. The results were analyzed using a two-tailed Student’s *t*-test (^∗^denotes *p* < 0.05, when compared to mock; center values are means and error bars represent s.e.m.).

To study the postnatal developmental impacts of congenital ZIKV infection in mouse pups (denoted ZIKV-pups), we inoculated pregnant immunocompetent C57BL/6J mice intraperitoneally (i.p.) at embryonic day (E) 8.5 with PBS (mock) or 10^4^ PFU of ZIKV strain PRVABC59, which is closely related to the epidemic strains that have been linked to human microcephaly in the Americas (Faria et al., 2016). Although at low levels, dams exposed to ZIKV developed viremia (**Supplementary Figure S1A**), confirming that both ZIKV can infect immunocompetent dams. Importantly, ZIKV RNA and infectious viral particles were detected in majority of the uteruses, placentas, and in fetal head and body tissues on day 2 post-infection (p.i.), indicating that ZIKV can infect the developing fetuses via the i.p. inoculation route (**Figures 1A,B**). In addition, maternal antibody- and type I interferon-mediated immunity (Miner et al., 2016; Zhao et al., 2016) (**Supplementary Figures S1B,C**) may have assisted in clearance of the virus, as ZIKV RNA was not detected in newborn ZIKV-pups brain tissue at birth (data not shown). Consistent with previous reports (Cugola et al., 2016; Miner et al., 2016), ZIKV- and mock-pups have comparable body weights at birth (**Figure 1C**), indicating that a mild congenital ZIKV infection in immunocompetent mice does not cause gross abnormalities at birth, in contrast to severe developmental and birth defects that were reported in pups born to ZIKV infected immunodeficient mice (Cugola et al., 2016; Miner et al., 2016). After birth, pups were monitored up to 17 weeks (120 days) to assess their growth, and any postnatal developmental and behavioral abnormalities. Interestingly, we found that ZIKV-pups gained less body weight than mock-pups approximately from D19 post-birth (p.b.) to the end of our experiments on D120 p.b. (**Figure 1C**), though ZIKV- and mock-infected dams have comparable body weights during both pregnancy and nursing periods (**Supplementary Figure S1D**) and gave birth to similar litters in terms of numbers and sex (data not shown). Furthermore, ZIKV-pups had statistically smaller head circumferences (**Figure 1D**) and smaller head and ear sizes (**Figure 1E**), than mock-pups. Collectively, these results suggested that a transient and mild congenital i.p. ZIKV infection could result in postnatal growth and developmental deficits in mouse pups, although no abnormalities were observed at birth.

**FIGURE 2:**
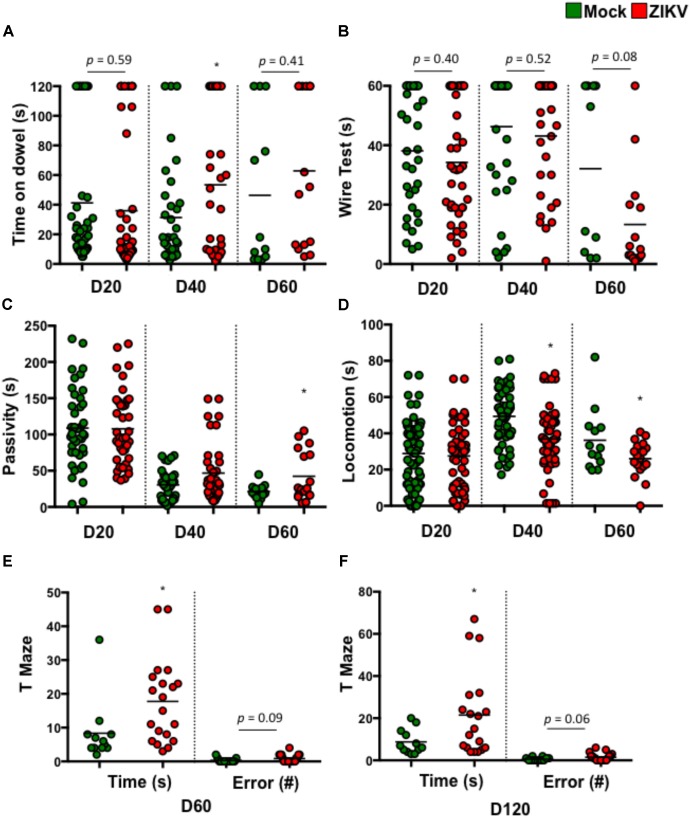
ZIKV-pups exhibit postnatal behavioral deficits. Pups born to dams infected with ZIKV (10^4^ PFU, i.p.) or mock infected on E8.5 were subjected to multiple behavioral tests. **(A)** Dowel test (s), *n* = 11–40; **(B)** Wire test (s), *n* = 11–40; **(C)** Bar cross test measurements of Passivity (s), *n* = 11–40; and **(D)** Locomotion (s), *n* = 11–40 and a T-Maze test of time to reward (s) and number of arm-entering errors (#) at D60 p.b. *n* = 11–19 **(E)**, and D120 p.b. *n* = 11–19 **(F)**. All behavioral measurements were blinded from the investigator handling the mice or the investigator performing the analysis. The results were compared using a two-tailed Student’s *t*-test (^∗^denotes *p* < 0.05, when compared to mock; center values are means).

To determine if these postnatal deficits were associated with neuron function, we assessed various neurodevelopmental behaviors in ZIKV- and mock-pups. These assessments included balance, motor coordination, forelimb strength, and cognitive development. The results showed that compared to mock-pups at selected time points p.b., ZIKV-pups had trends in poor balance (**Figure 2A**) and weak fore-limb strength (**Figure 2B**), suggesting motor coordination disparities. Along with a significant increase in passivity and decrease in locomotion, indicating a deficit in motor or motivational behavior (**Figures 2C,D**). While, the cognitive memory T-maze test indicated a significant increase in the time it took to enter a reward-containing arm, with a trend in increased errors made to enter the same arm (**Figures 2E,F**). These behavior results indicated that even a mild congenital ZIKV infection could cause postnatal neurobehavioral defects, especially related to cognitive function. In support of these tests, a visual cliff recognition test indicated that ZIKV-pups were inclined to make poor decisions compared to mock-pups particularly in adulthood (**Supplementary Figure S2A**). In addition, ocular analyses displayed normal histology of the cornea, lens, angle, ciliary body and retina, indicating that the neurobehavioral deficits in ZIKV-pups might not be due to ocular defects in our model (**Supplementary Figure S2B**). Since in this model we intended to mimic a mild ZIKV infection in the majority of human infection cases by inoculating a low dose of ZIKV via i.p. in immunocompetent mice, it is possible that the viral infection levels in the fetuses varies (**Figures 1A,B**), which may explain why some ZIKV-pups manifested only mild neurobehavioral deficits, or even similar behaviors compared to mock-pups. We speculated that adjustment of the viral infection doses along with inoculation timing during embryonic development might affect the degree of postnatal neurobehavioral deficits. Nevertheless, our results indicated that a mild ZIKV infection *in utero* could cause both postnatal motor and memory impairments that persisted into adulthood.

**FIGURE 3:**
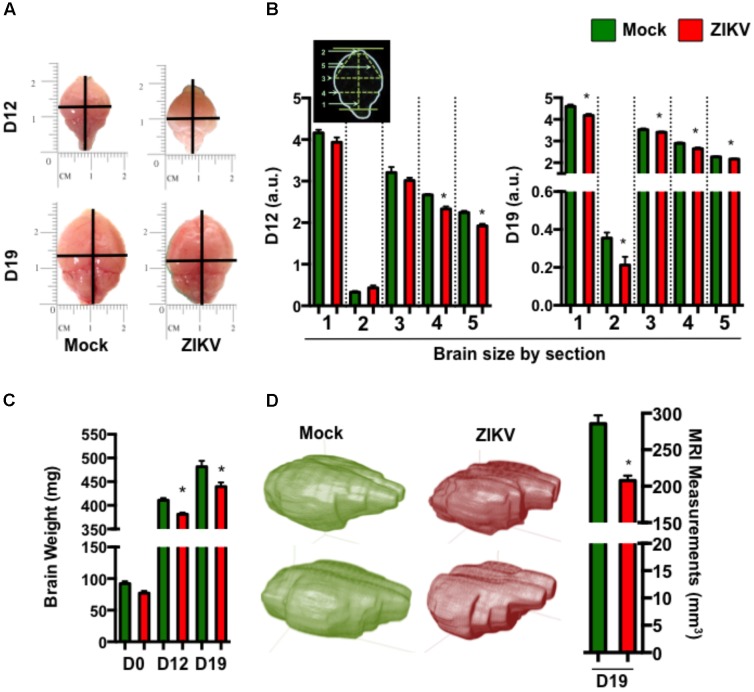
ZIKV-pups have smaller brains. Brains from pups born to dams infected with ZIKV (10^4^ PFU, i.p.) or mock infected on E8.5 were analyzed. **(A)** Representative brain images at D12 and D19 p.b. **(B)** Metric measurement of scaled-up images for resolution (a.u. denotes arbitrary units) of brain sections: “1,” length of brain (midline) from top of olfactory bulb to bottom of cerebellum; “2,” length of olfactory bulb; “3,” width of cerebrum; “4,” width of bottom of cerebrum; “5,” length from midline of olfactory bulb (bottom) to side of cerebrum, at D12 p.b., *n* = 8 and D19 p.b., *n* = 13–16. **(C)** Weight (mg) of brains (D0 p.b., *n* = 9–17; D12 p.b., *n* = 13–14; D19 p.b., *n* = 17–20). **(D)** 3D images and volume measurement (mm^3^) of brains from pups (*n* = 2) at D19 p.b. obtained by MRI. The brain measurements and weights were compared using a two-tailed Student’s *t*-test and the MRI measurements were compared using a Mann–Whitney *U* test (^∗^denotes *p* < 0.05; center values are means and error bars represent s.e.m).

**FIGURE 4:**
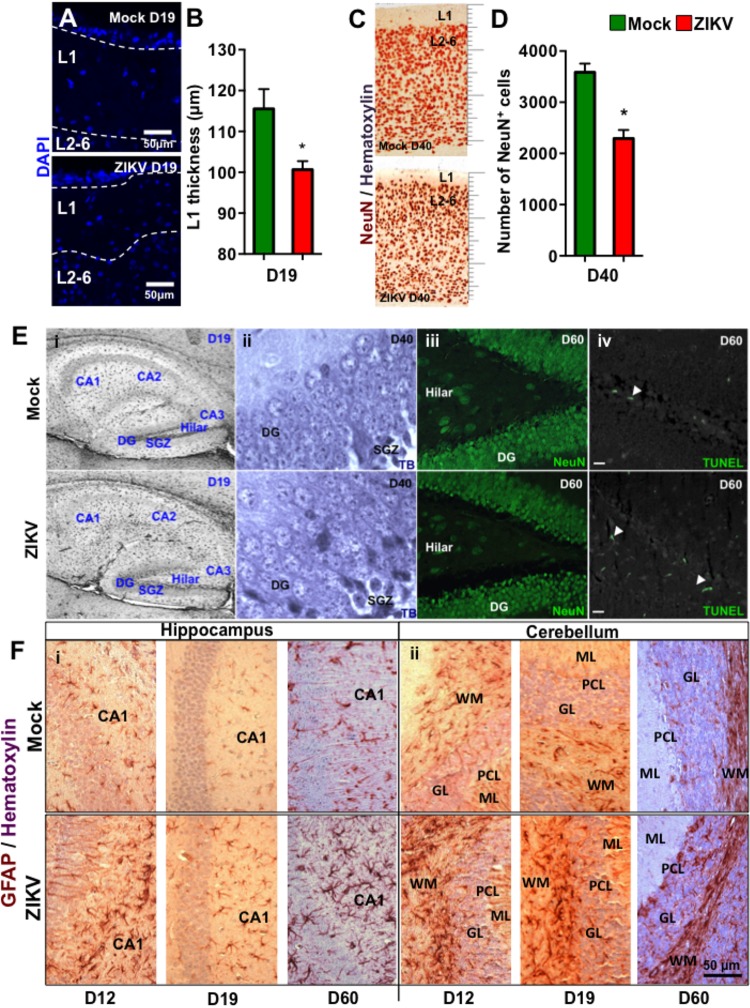
Congenital ZIKV infection causes a thinner cortical layer 1, dysregulated neuronal migration and astrogliosis. Brain sections of pups from ZIKV (10^4^ PFU, i.p.) or mock-infected dams on E8.5 were analyzed at various time points post-birth. **(A)** Representative images of cortical layers (L1-6) of D19 p.b. coronal brain sections stained with DAPI. **(B)** Layer 1 (L1) thickness measurements per 100× magnification field were quantified by using ImageJ software (*n* = 3). **(C)** Representative images of brains (D40 p.b.) stained with NeuN showing cortical layers 1–6 (L1-6, 400× magnification). **(D)** Quantification of NeuN^+^ cells in the coronal cortical sections per 400× magnification field at D40 p.b. (*n* = 3). **(E)** Anatomical images of **(i)** hippocampus at D19 p.b. (80× magnification); **(ii)** dentate gyrus at D40 p.b. (1000× magnification); **(iii)** NeuN^+^ dentate gyrus and hilar region of the hippocampus (400× magnification); and **(iv)** TUNEL labeled dentate gyrus and hilar region of the hippocampus (400× magnification, scale bar = 20 μm). **(F)** GFAP immunoreactivity (brown) and hematoxylin (purple) staining of midsagittal sections (D12, D19, and D60 p.b.) of the hippocampus CA1 labeled neurons (**i,** left panel); and the cerebellum (**ii,** right panel). DG, dentate gyrus; SGZ, subgranular zone; ML, molecular layer; PCL, Purkinje cell layer; GL, granule cell layer; WM, white matter. Images represent three biological samples from each group. The L1 thickness and the number of the NeuN^+^ cells were compared using a Mann–Whitney *U* test (^∗^denotes *p* < 0.05; center values are means and error bars represent s.e.m.).

To further characterize the effect of congenital ZIKV infection on postnatal brain development, we compared brain weights and sizes of ZIKV- and mock-pups on D0, D12 and D19 p.b. Although ZIKV-pups had comparable brain weights with mock-pups on D0, their brain weights and sizes were smaller than mock-pups on D12 and D19 p.b. (**Figures 3A–C**). Furthermore, three-dimensional (3D) magnetic resonance imaging (MRI) of whole brains on D19 p.b. revealed that ZIKV-pups had smaller brain volumes compared to mock-pups (**Figure 3D**). In addition, immunostaining of midsagittal brain sections showed cellular disarrangement and a thinner cortical layer 1 in the brains of ZIKV-pups at D19 p.b. (**Figures 4A,B**), a feature associated with microcephaly in human babies (Li C. et al., 2016). In line with this, calcium-binding protein Calbindin-D28k positive neurons migrated into the layer 1 in the brains of ZIKV-pups (**Supplementary Figure S3C**), which are usually seen in layers four to six (L4-6) (Copp and Harding, 1999). It is important to note that cortical layer 1 formation in mouse fetal brain occurs between E8-10 of gestation (Copp and Harding, 1999), a time window that is similar to the first trimester of human pregnancy, whereby the developing fetus is most susceptible to develop ZIKV-mediated neurological deficits (Johansson et al., 2016). Apart from cortical layer 1 thinness in ZIKV-pups at D40 p.b., the density of neurons within L1-6 of the cortex were also reduced (**Figures 4C,D**). Since memory deficits were observed in adult ZIKV-pups, but not in mock-pups, we next analyzed whole brain pathology of ZIKV-pups, which indicated differential cellular architecture, particularly within the CA1 and dentate gyrus regions of the hippocampus (**Figures 4Ei–iii**). Interestingly, the subgranular zone of the dentate gyrus is the site for neurogenesis (Eriksson et al., 1998), and neural progenitor cells within this region are highly susceptible to ZIKV infection, resulting in cellular apoptosis (Li H. et al., 2016; Wu et al., 2016). In comparison, both reduced neurons and marginal apoptosis were observed in the dentate gyrus region of the hippocampus in ZIKV-pups up to adulthood (**Figures 4Eiii,iv**), along with the neocortex (**Supplementary Figures S3A,B**) and the cerebellum (data not shown), suggesting that developmental memory deficits may be a result of disrupted postnatal neurogenesis and neural function. Furthermore, midsagittal brain sections were immunostained with GFAP to identify astrogliosis, a hallmark feature of human and mouse newborns with microcephaly (Mlakar et al., 2016; Shao et al., 2016). The GFAP immunostaining exhibited progressive astrogliosis near CA1 neurons of the hippocampus and within the white matter of the cerebellum of ZIKV-pups at D12, D19 and D60 p.b. (**Figure 4F**), along with enlarged astrocytes surrounding motor neurons within cervical spinal cords (**Supplementary Figure S3D**), while only minimal reactivity to GFAP was detected in these regions in mock-pups (**Figure 4F** and **Supplementary Figure S3D**). Reactive astrogliosis in the hippocampus and cerebellum has been linked to defective memory (Shipton et al., 2014) and motor coordination (Cendelin, 2014) in mice, which were observed in our behavioral studies of ZIKV-pups (**Figures 2A–F** and **Supplementary Figure S2A**). Consistent with our report, chronic astrogliosis, neurodegeneration and reduced cognitive neurobehavioral performances have also been observed following murine cytomegalovirus infection, even after the virus was cleared (Mutnal et al., 2011; Lokensgard et al., 2015). Collectively, these observations suggested that a transient and mild ZIKV infection in immunocompetent mice during pregnancy could cause postnatal brain developmental deficits in pups.

**FIGURE 5:**
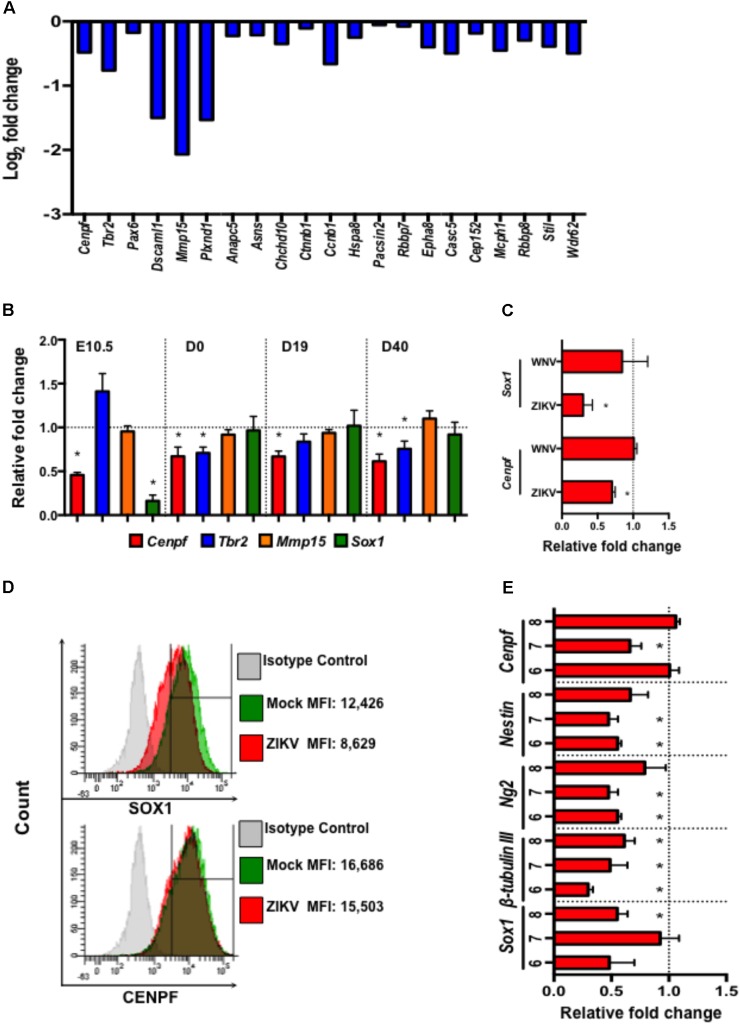
Zika virus down-regulates the expression of neural development- and microcephaly- related genes in Neuro-2a cells, ZIKV-pup brain tissue, and embryoid bodies. **(A)** Expression profile of neural development- and microcephaly- related genes in ZIKV-infected Neuro-2a cells by mRNA sequencing. **(B)** qPCR measurement of the expression of neuronal- and microcephaly-related genes in fetal brains on E10.5 (*n* = 12/group), and in pup brains on D0, D19, and D40 p.b. (*n* = 7–13/group). **(C)** qPCR analysis of the expression of *Sox1* and *Cenpf* in Neuro-2a cells infected with ZIKV and WNV, (MOI = 1) for 24 h. **(D)** Flow cytometric analysis of SOX1 and CENPF expression in Neuro-2a cells infected with ZIKV (MOI = 1) for 48 h. MFI, mean fluorescence intensity. **(E)** Expression of neural development- and microcephaly- related genes in embryoid bodies (EB) (6–8 days old) infected with ZIKV (10^4^ PFU). qPCR analyses are represented as relative fold change (RFC) and are normalized to cellular *β-actin*, with a definition of mock control as 1. Data were compared using a two-tailed, Student’s *t*-test (^∗^denotes *p* < 0.05; error bars represents s.e.m.). qPCR and flow cytometry experiments were repeated twice.

Zika virus, as a neurotropic virus, preferentially targets neural stem cells and immature neurons (Hughes et al., 2016; Nowakowski et al., 2016). Therefore, we also studied the impact of ZIKV infection on a global gene expression profile in murine neuroblasts (Neuro-2a) by mRNA sequencing (RNA-seq). Differential expression analysis revealed down-regulation of several neural development- and microcephaly- related genes including *Cenpf, Tbr2, Pax6, Dscaml1, Mmp15, Casc5, Rbbp8, Mcph1, Stil, and Wdr62* (**Figure 5A**). While similar neural developmental- and microcephaly- related genes were also measured in fetal head tissues and pups’ brains by quantitative real-time PCR (qPCR). The results showed that the expression of *Cenpf* and the neurogenesis gene, *Sox1* (Venere et al., 2012), were reduced in the head tissue of ZIKV-infected fetuses on E10.5 (**Figure 5B**). Moreover, *Cenpf* (D0, 19, and 40 p.b.) and *Tbr2* (D0 and 40 p.b.) were significantly down-regulated in brains of ZIKV-pups compared to mock-pups, with no differences detected in *Mmp15* expression (**Figure 5B**). In humans, mutations in *Cenpf* are associated with congenital malformation syndromes and microcephaly (Waters et al., 2015; Filges et al., 2016). Similarly, silencing of transcription factor Eomes/Tbr2 during early fetal development could lead to disorders of neuronal migration, microcephaly (Baala et al., 2007; Arnold et al., 2008), and behavioral deficits, including reduced hang wire strength of mouse pups (Arnold et al., 2008), with this trend being observed in our ZIKV-pups (**Figure 2B**). Since the transcription of *Cenpf and Sox1* were reduced in the head tissue of ZIKV-infected fetuses on E10.5 (**Figure 5B**), we further confirmed down-regulation by qPCR analysis in Neuro-2a cells infected with ZIKV, while no difference was observed when cells were infected with another closely related flavivirus, West Nile virus (WNV, strain CT2741) (**Figure 5C**). Down-regulation of SOX1 and CENPF by ZIKV was further confirmed in ZIKV-infected Neuro-2a cells by flow cytometry (**Figure 5D**). Murine embryonic stem cells (ESCs) have the potential to differentiate into neuronal precursors, therefore, we sought to evaluate the effects of ZIKV infection on pre-neuronal development in our established *in vitro* ESC model (Guo et al., 2007). When ESCs are cultured in suspension, they grow into aggregates, forming embryoid bodies (EBs) and 3D structures that resemble an early embryo, whereby neurons and neuronal progenitors can be readily detected (Guo et al., 2007). In this model, we infected 5-day-old EBs with ZIKV to assess the expression of neurodevelopment- and microcephaly related genes, including *Sox1*, *β-tubulin III, Ng2*, *Nestin*, and *Cenpf* on days 6-8 (D6-8) by qPCR. The results also showed that ZIKV infection down-regulated the expression of multiple neuron-specific genes at a time frame that resembles early stages of embryogenesis (**Figure 5E**). Although more detailed research is warranted to define the roles of these genes in ZIKV infection, the altered expression of the microcephaly-related genes in the fetal brains may directly or indirectly result in the neuronal pathology, postnatal growth impediment, and neurobehavioral deficits in the ZIKV-pups.

Congenital ZIKV infection in humans causes severe microcephaly at birth only in a small percent of babies (Cauchemez et al., 2016; Johansson et al., 2016; Reynolds et al., 2017), while some apparently healthy newborn babies can still develop postnatal congenital ZIKV syndromes including head growth restrictions and behavioral deficits, before 1 year of age (Bilbo and Schwarz, 2009; Felix et al., 2017). Consistent with our findings in mice, some human infants, without apparent microcephaly at birth, displayed postnatal syndromes of disproportionate head growth, while one baby developed difficulties moving its left hand months after birth. Not surprisingly, neuroimaging analyses suggested that babies who developed postnatal congenital ZIKV syndromes had a milder degree of brain damage compared to those with microcephaly at birth (Aragao et al., 2017). Our ZIKV infection model in immunocompetent C57BL/6J mice causes brain widespread astrogliosis and mild neurobehavioral deficits in pups, which can partially mimic congenital ZIKV-causing postnatal developmental deficits in humans. In addition, ZIKV-pups developed cognitive deficits by measurement of the T-maze test that persisted into adulthood indicating even a mild congenital ZIKV infection could result in learning disabilities in humans.

While our current experimental design has some limitations, such as low viral RNA infection levels that were not detected in all fetal samples, our mouse model may mimic majority of human ZIKV infections. This mouse model can be further optimized by adjusting infection timing and increasing viral dose in that ZIKV-pups may display more robust neurobehavioral deficits, making this a valuable animal model to mimic human congenital ZIKV-caused postnatal developmental deficits. Although we cannot exclude the possibility that *in utero* inflammation may also contribute to the developmental deficits of ZIKV-pups, our results are consistent with other congenital viral infections, such as CMV and rubella, which can both cause postnatal manifestations in human infants (Sever et al., 1985; Fowler et al., 1992), suggesting ZIKV infection during pregnancy could also lead to postnatal developmental deficits that may due to direct or indirect damages of ZIKV infection in neural progenitor cells.

In conclusion, our results showed that even a transient, mild congenital ZIKV infection in immunocompetent mice could lead to postnatal neurobehavioral deficits, suggesting it is necessary to closely monitor both physical and intellectual development in children whose mothers were exposed to ZIKV infection during pregnancy. In addition, our study also provides a valuable animal model to mimic human congenital ZIKV-infection caused postnatal developmental deficits.

## Materials and Methods

### Ethics Statement and Biosafety

All animal care and experiments were conducted according to the Guide for the Care and Use of Laboratory Animals approved by The University of Southern Mississippi (USM) under the IACUC protocol # 16031002. All *in vitro* experiments and animal studies involving live ZIKV and WNV were performed by certified personnel in biosafety level 2 and 3 laboratories following standard biosafety protocols approved by the USM Institutional Biosafety Committees.

### Viruses, Animals, and Cells

Zika virus (strain PRVABC59) was obtained from B. Johnson (CDC Arbovirus Branch, Fort Collins, CO, United States) and WNV isolate (CT2741), kindly provided by John F. Anderson, were propagated in Vero cells (ATCC CCL-81). Viral stocks were titered in Vero cells by a plaque assay, as previously described (Paul et al., 2014). C57BL/6J mice were purchased from the Jackson Laboratory (Bar Harbor, ME, United States) and 9–10 week-old mice were paired for copulation. When vaginal plugs appeared (embryonic day, E0.5) pairs were separated. At E8.5, dams were inoculated i.p. with ZIKV (10^4^ PFU), or phosphate buffered saline (PBS) as mock control. Murine neuroblast cells (Neuro-2a, ATCC CCL-131) were cultured in DMEM supplemented with 10% FBS and 1% Pen/Strep at 37°C with 5% CO_2._ Mouse ESCs (D3 cell line, ATCC) were maintained in mouse ESC medium. ESC differentiation through embryoid body (EB) formation was performed as previously described (Guo et al., 2007).

### Quantitative PCR (qPCR) and Plaque-Forming Unit (PFU) Assay

Total RNA was extracted from tissues or cultured cells by using TRIreagent (Molecular Research Center, Inc.) and converted to cDNA using iSCRIPT cDNA synthesis kit (Bio-Rad). QPCR assays were performed using iTAQ polymerase supermix for probe-based assays (Bio-Rad) or iQ SYBR Green Supermix (Bio-Rad). Viral RNA of ZIKV *envelope (E)* (Acharya et al., 2016) was measured by qPCR and infectious viruses were measured by plaque assay as we previously described (Acharya et al., 2015). Threshold cycle values that were ≥39 cycles were excluded from the qPCR results, and 1 PFU per volume of sample was set as the limit of viral detection for the plaque assays. WNV-*envelope* (*E)* gene primers and probes sequences were adapted according to a previous publication (Town et al., 2009). All additional gene primer sequences are described in **Supplementary Table S1**.

### Neurobehavioral Tests

#### Dowel Test

Balance and motor coordination was measured using a 24-inch long dowel (0.9 cm in diameter), attached to a bar cross apparatus and the time mice remained on the dowel was recorded for up to a maximum of 2 min, the longer time that mice remained on the dowel indicated balance/motor coordination disparity (Vig et al., 2012).

#### Wire Suspension Test

As previously described (Santos et al., 2007), mice were hung onto a 3-mm wire with their forelimbs for 1 min and the time they took to fall (seconds) was recorded.

#### Bar Cross Test

Mice were placed on one arm of a U-shaped bar cross apparatus (30 cm high and 18 mm wide) and allowed to move for 5 min. Locomotion time (duration of mobile activity) and passivity time (duration of total inactivity) was recorded (Vig et al., 2012).

#### T-Maze Test

Mice were habituated (5 min/mouse) in a T-maze with dimensions previously described (Deacon and Rawlins, 2006), for 5 days prior to testing. A side preference was noted during habituation days and on the test day; a mouse was placed in the starting arm, with the preferred arm closed and the other arm open (alternating arm test), with both arms containing a reward (Kellog’s^®^ Fruitloops^®^). Once the mouse explored the maze by finding the reward and returning to the start arm, the closed arm was opened and the timer was started. The time it took (seconds) and the number or errors (entering incorrect arm) was recorded.

All the neurobehavioral tests were blinded to both the investigator performing the tests and another investigator collecting data.

### Magnetic Resonance Imaging

Whole heads were collected on D19 p.b., fixed in 4% PFA for 96 h, and transferred to PBS until ready for MRI imaging. All MRI images were acquired with a GE Signa Excite HDx MR machine with a 3.0 T magnet. The skulls were individually placed into the Mayo Clinic BC-10 Wrist Coil (Part #13G5614) and imaged using a Transverse T2 fast spin echo sequence. Sequence parameters included an 85 ms echo time (TE), 3350 ms repetition time (TR), 4 cm field of view, 12 echo train length (ETC), and 3 averages, with 1.0 mm slice thickness and 0.0 mm slice spacing. Image segmentation, 3D reconstruction, and dimensional quantifications were performed using Scan IP (Simpleware, United Kingdom).

### Immunohistochemistry and Immunofluorescence

Midsagittal and coronal brain sections (6 μm) at D12, D19, D40, and D60 p.b were probed with NeuN and GFAP specific antibodies, followed by labeling with Alexa 488 secondary antibody (Invitrogen) and DAPI and TUNEL for immunofluorescence, or biotinylated secondary antibody, developed with ExtrAvidin peroxidase immunostaining kit (Sigma), for immunohistochemistry. Non-specific Toluidine blue and hematoxylin staining was also performed. The slides were mounted with Aqua-mount (Fisher Scientific) and observed using an Epi-fluorescence (Olympus BX60) or bright-field microscope, and images were captured using a digital camera (DP70).

### Flow Cytometry

Neuro-2a cells infected with ZIKV (MOI = 1) for 48 h were fixed in 4% PFA, probed with rabbit anti-CENPF or anti-SOX-1 antibodies (Abcam), followed by FITC-conjugated goat-anti-rabbit-IgG antibody (Santa Cruz). Cells were then washed twice and analyzed with a BD LSRFortessa flow cytometer (BD Biosciences) and data were acquired using the BD FACSDIVA^TM^ version 7.0 (BD Biosciences). Cells probed with secondary IgG antibodies were used as fluorescence gating controls.

### RNA-Sequencing (RNA-seq)

Neuro-2a cells were infected with ZIKV (MOI = 0.5) for 48 h and total RNA was extracted and purified using TRI Reagent and RNeasy Mini kit (Qiagen). RNA-seq was performed at the Molecular and Genomics Core Facility of the University of Mississippi Medical Center.

### Statistical Analyses

Data were compared with a two-tailed Student’s *t*-test or a Mann-Whitney U test GraphPad Prism software (version 6.0), with *p* < 0.05 considered statistically significant.

## Data Availability

RNA-seq results have been deposited in the NCBI BioProject database (Accession: PRJNA385324). All the other data supporting the findings of this study are available within the article and its Supplementary Information files, or are available from the authors upon request.

## Author Contributions

FB conceived the experiments. AP conducted most of the experiments. DA, BN, ET, GG-F, KC, MG, ML, FG-F, and PV assisted in experiments and analyzed the results. HL helped with statistical analysis. FB and AP wrote the manuscript. MC, AF, JL, and Y-LG provided experimental materials. All the authors read and approved the manuscript.

## Conflict of Interest Statement

The authors declare that the research was conducted in the absence of any commercial or financial relationships that could be construed as a potential conflict of interest.

## References

[B1] Acharya D. Bastola P. Le L. Paul A. M. Fernandez E. Diamond M. S. (2016). An ultrasensitive electrogenerated chemiluminescence-based immunoassay for specific detection of Zika virus. *Sci. Rep.* 6:32227. 10.1038/srep32227 27554037 PMC4995374

[B2] Acharya D. Paul A. M. Anderson J. F. Huang F. Bai F. (2015). Loss of glycosaminoglycan receptor binding after mosquito cell passage reduces chikungunya virus infectivity. *PLoS Negl. Trop. Dis.* 9:e0004139. 10.1371/journal.pntd.0004139 26484530 PMC4615622

[B3] Aragao M. F. V. V. Holanda A. C. Brainer-Lima A. M. Petribu N. C. L. Castillo M. van der Linden V. (2017). Nonmicrocephalic infants with congenital Zika syndrome suspected only after neuroimaging evaluation compared with those with microcephaly at birth and postnatally: how large is the Zika virus “Iceberg”? *AJNR Am. J. Neuroradiol.* 38 1427–1434. 10.3174/ajnr.A5216 28522665 PMC7959892

[B4] Arnold S. J. Huang G. J. Cheung A. F. Era T. Nishikawa S. Bikoff E. K. (2008). The T-box transcription factor Eomes/Tbr2 regulates neurogenesis in the cortical subventricular zone. *Genes Dev.* 22 2479–2484. 10.1101/gad.475408 18794345 PMC2546697

[B5] Baala L. Briault S. Etchevers H. C. Laumonnier F. Natiq A. Amiel J. (2007). Homozygous silencing of T-box transcription factor EOMES leads to microcephaly with polymicrogyria and corpus callosum agenesis. *Nat. Genet.* 39 454–456. 10.1038/ng1993 17353897

[B6] Bilbo S. D. Schwarz J. M. (2009). Early-life programming of later-life brain and behavior: a critical role for the immune system. *Front. Behav. Neurosci.* 3:14. 10.3389/neuro.08.014.2009 19738918 PMC2737431

[B7] Campos G. S. Bandeira A. C. Sardi S. I. (2015). Zika Virus outbreak, Bahia, Brazil. *Emerg. Infect. Dis.* 21 1885–1886. 10.3201/eid2110.150847 26401719 PMC4593454

[B8] Carteaux G. Maquart M. Bedet A. Contou D. Brugières P. Fourati S. (2016). Zika virus Associated with meningoencephalitis. *N. Engl. J. Med.* 374 1595–1596. 10.1056/NEJMc1602964 26958738

[B9] Cauchemez S. Besnard M. Bompard P. Dub T. Guillemette-Artur P. Eyrolle-Guignot D. (2016). Association between Zika virus and microcephaly in French Polynesia, 2013-15: a retrospective study. *Lancet* 387 2125–2132. 10.1016/S0140-6736(16)00651-6 26993883 PMC4909533

[B10] Cendelin J. (2014). From mice to men: lessons from mutant ataxic mice. *Cerebellum Ataxias* 1:4. 10.1186/2053-8871-1-4 26331028 PMC4549131

[B11] Copp A. J. Harding B. N. (1999). Neuronal migration disorders in humans and in mouse models–an overview. *Epilepsy Res.* 36 133–141. 10515161 10.1016/s0920-1211(99)00047-9 PMC4471133

[B12] Cugola F. R. Fernandes I. R. Russo F. B. Freitas B. C. Dias J. L. M. Guimarães K. P. (2016). The Brazilian Zika virus strain causes birth defects in experimental models. *Nature* 534 267–271. 10.1038/nature18296 27279226 PMC4902174

[B13] Cui L. Zou P. Chen E. Yao H. Zheng H. Wang Q. (2017). Visual and Motor Deficits in Grown-up Mice with Congenital Zika Virus Infection. *EBioMedicine* 20 193–201. 10.1016/j.ebiom.2017.04.029 28583742 PMC5478201

[B14] Dang J. Tiwari S. K. Lichinchi G. Qin Y. Patil V. S. Eroshkin A. M. (2016). Zika virus depletes neural progenitors in human cerebral organoids through activation of the innate immune receptor TLR3. *Cell Stem Cell* 19 258–265. 10.1016/j.stem.2016.04.014 27162029 PMC5116380

[B15] Deacon R. M. Rawlins J. N. (2006). T-maze alternation in the rodent. *Nat. Protoc.* 1 7–12. 10.1038/nprot.2006.2 17406205

[B16] D’Ortenzio E. Matheron S. Yazdanpanah Y. de Lamballerie X. Hubert B. Piorkowski G. (2016). Evidence of Sexual Transmission of Zika Virus. *N. Engl. J. Med.* 374 2195–2198. 10.1056/NEJMc1604449 27074370

[B17] Eriksson P. S. Perfilieva E. Björk-Eriksson T. Alborn A. M. Nordborg C. Peterson D. A. (1998). Neurogenesis in the adult human hippocampus. *Nat. Med.* 4 1313–1317. 10.1038/3305 9809557

[B18] Faria N. R. Azevedo R. D. S. D. S. Kraemer M. U. G. Souza R. Cunha M. S. Hill S. C. (2016). Zika virus in the Americas: early epidemiological and genetic findings. *Science* 352 345–349. 10.1126/science.aaf5036 27013429 PMC4918795

[B19] Felix A. Hallet E. Favre A. Kom-Tchameni R. Defo A. Fléchelles O. (2017). Cerebral injuries associated with Zika virus in utero exposure in children without birth defects in French Guiana: case report. *Medicine* 96:e9178. 10.1097/MD.0000000000009178 29390455 PMC5758157

[B20] Filges I. Bruder E. Brandal K. Meier S. Undlien D. E. Waage T. R. (2016). Stromme syndrome is a ciliary disorder caused by mutations in CENPF. *Hum. Mutat* 37 359–363. 10.1002/humu.22960 26820108

[B21] Fowler K. B. Stagno S. Pass R. F. Britt W. J. Boll T. J. Alford C. A. (1992). The outcome of congenital cytomegalovirus infection in relation to maternal antibody status. *N. Engl. J. Med.* 326 663–667. 10.1056/NEJM199203053261003 1310525

[B22] Garcez P. P. Loiola E. C. Madeiro da Costa R. Higa L. M. Trindade P. Delvecchio R. (2016). Zika virus impairs growth in human neurospheres and brain organoids. *Science* 352 816–818. 10.1126/science.aaf6116 27064148

[B23] Grant A. Ponia S. S. Tripathi S. Balasubramaniam V. Miorin L. Sourisseau M. (2016). Zika Virus Targets Human STAT2 to Inhibit Type I Interferon Signaling. *Cell Host Microbe* 19 882–890. 10.1016/j.chom.2016.05.009 27212660 PMC4900918

[B24] Guo Y. L. Ye J. Huang F. (2007). p38alpha MAP kinase-deficient mouse embryonic stem cells can differentiate to endothelial cells, smooth muscle cells, and neurons. *Dev. Dyn* 236 3383–3392. 10.1002/dvdy.21374 17994546

[B25] Hayes E. B. (2009). Zika virus outside Africa. *Emerg. Infect. Dis.* 15 1347–1350. 10.3201/eid1509.090442 19788800 PMC2819875

[B26] Hirsch A. J. Roberts V. H. J. Grigsby P. L. Haese N. Schabel M. C. Wang X. (2018). Zika virus infection in pregnant rhesus macaques causes placental dysfunction and immunopathology. *Nat. Commun.* 9:263. 10.1038/s41467-017-02499-9 29343712 PMC5772047

[B27] Hughes B. W. Addanki K. C. Sriskanda A. N. McLean E. Bagasra O. (2016). Infectivity of immature neurons to zika virus: a link to congenital zika syndrome. *EBioMedicine* 10 65–70. 10.1016/j.ebiom.2016.06.026 27364784 PMC5006602

[B28] Johansson M. A. Mier-y-Teran-Romero L. Reefhuis J. Gilboa S. M. Hills S. L. (2016). Zika and the Risk of Microcephaly. *N. Engl. J. Med.* 375 1–4. 10.1056/NEJMp1605367 27222919 PMC4945401

[B29] Lazear H. M. Govero J. Smith A. M. Platt D. J. Fernandez E. Miner J. J. (2016). A Mouse Model of Zika Virus Pathogenesis. *Cell Host Microbe* 19 720–730. 10.1016/j.chom.2016.03.010 27066744 PMC4866885

[B30] Li C. Xu D. Ye Q. Hong S. Jiang Y. Liu X. (2016). Zika virus disrupts neural progenitor development and leads to microcephaly in mice. *Cell Stem Cell* 19 120–126. 10.1016/j.stem.2016.04.017 27179424

[B31] Li H. Saucedo-Cuevas L. Regla-Nava J. A. Chai G. Sheets N. Tang W. (2016). Zika virus infects neural progenitors in the adult mouse brain and alters proliferation. *Cell Stem Cell* 19 593–598. 10.1016/j.stem.2016.08.005 27545505 PMC5097023

[B32] Licon A. (2016). *Zika ‘syndrome’: Health Problems Mount as Babies Turn 1*. Available at: http://bigstory.ap.org/article/e8162d68647643faa3066a366b6da5bb/babies-stricken-zika-turn-1-health-problems-mount

[B33] Lokensgard J. R. Schachtele S. J. Mutnal M. B. Sheng W. S. Prasad S. Hu S. (2015). Chronic reactive gliosis following regulatory T cell depletion during acute MCMV encephalitis. *Glia* 63 1982–1996. 10.1002/glia.22868 26041050 PMC4670295

[B34] Miner J. J. Cao B. Govero J. Smith A. M. Fernandez E. Cabrera O. H. (2016). Zika virus infection during pregnancy in mice causes placental damage and fetal demise. *Cell* 165 1081–1091. 10.1016/j.cell.2016.05.008 27180225 PMC4874881

[B35] Mlakar J. Korva M. Tul N. Popović M. Poljšak-Prijatelj M. Mraz J. (2016). Zika virus associated with microcephaly. *N. Engl. J. Med.* 374 951–958. 10.1056/NEJMoa1600651 26862926

[B36] Mounce B. C. Poirier E. Z. Passoni G. Simon-Loriere E. Cesaro T. Prot M. (2016). Interferon-induced spermidine-spermine acetyltransferase and polyamine depletion restrict Zika and chikungunya viruses. *Cell Host Microbe* 20 167–177. 10.1016/j.chom.2016.06.011 27427208

[B37] Mutnal M. B. Hu S. Little M. R. Lokensgard J. R. (2011). Memory T cells persisting in the brain following MCMV infection induce long-term microglial activation via interferon-gamma. *J. Neurovirol.* 17 424–437. 10.1007/s13365-011-0042-5 21800103 PMC3204167

[B38] Nowakowski T. J. Pollen A. A. Di Lullo E Sandoval-Espinosa C. Bershteyn M. Kriegstein A. R. (2016). Expression analysis highlights AXL as a Candidate Zika virus entry receptor in neural stem cells. *Cell Stem Cell* 18 591–596. 10.1016/j.stem.2016.03.012 27038591 PMC4860115

[B39] Oehler E. Watrin L. Larre P. Leparc-Goffart I. Lastere S. Valour F. (2014). Zika virus infection complicated by Guillain-Barre syndrome–case report, French Polynesia, December 2013. *Euro Surveill.* 19 20720. 10.2807/1560-7917.es2014.19.9.20720 24626205

[B40] Paul A. M. Shi Y. Acharya D. Douglas J. R. Cooley A. Anderson J. F. (2014). Delivery of antiviral small interfering RNA with gold nanoparticles inhibits dengue virus infection in vitro. *J. Gen. Virol.* 95 1712–1722. 10.1099/vir.0.066084-0 24828333 PMC4103068

[B41] Pessoa A. van der Linden V. Yeargin-Allsopp M. Carvalho M. D. C. G. Ribeiro E. M. Van Naarden Braun K. (2018). Motor Abnormalities and Epilepsy in Infants and Children With Evidence of Congenital Zika Virus Infection. *Pediatrics* 141 S167–S179. 10.1542/peds.2017-2038F 29437050

[B42] Reynolds M. R. Jones A. M. Petersen E. E. Lee E. H. Rice M. E. Bingham A. (2017). Vital signs: update on zika virus-associated birth defects and evaluation of All U.S. Infants with congenital Zika virus exposure - U.S. Zika pregnancy registry, 2016. *MMWR Morb. Mortal. Wkly. Rep.* 66 366–373. 10.15585/mmwr.mm6613e1 28384133 PMC5657905

[B43] Rossi S. L. Tesh R. B. Azar S. R. Muruato A. E. Hanley K. A. Auguste A. J. (2016). Characterization of a novel murine model to study Zika virus. *Am. J. Trop. Med. Hyg.* 94 1362–1369. 10.4269/ajtmh.16-0111 27022155 PMC4889758

[B44] Santos M. Silva-Fernandes A. Oliveira P. Sousa N. Maciel P. (2007). Evidence for abnormal early development in a mouse model of Rett syndrome. *Genes Brain Behav.* 6 277–286. 10.1111/j.1601-183X.2006.00258.x 16848781

[B45] Sever J. L. South M. A. Shaver K. A. (1985). Delayed manifestations of congenital rubella. *Rev. Infect. Dis.* 7(Suppl. 1), S164–S169. 4001724 10.1093/clinids/7.supplement_1.s164

[B46] Shao Q. Herrlinger S. Yang S. L. Lai F. Moore J. M. Brindley M. A. (2016). Zika virus infection disrupts neurovascular development and results in postnatal microcephaly with brain damage. *Development* 143 4127–4136. 10.1242/dev.143768 27729407 PMC5117220

[B47] Shipton O. A. El-Gaby M. Apergis-Schoute J. Deisseroth K. Bannerman D. M. Paulsen O. (2014). Left-right dissociation of hippocampal memory processes in mice. *Proc. Natl. Acad. Sci. U.S.A.* 111 15238–15243. 10.1073/pnas.1405648111 25246561 PMC4210314

[B48] Tabata T. Petitt M. Puerta-Guardo H. Michlmayr D. Wang C. Fang-Hoover J. (2016). Zika Virus Targets Different Primary Human Placental Cells, Suggesting Two Routes for Vertical Transmission. *Cell Host Microbe* 20 155–166. 10.1016/j.chom.2016.07.002 27443522 PMC5257282

[B49] Tang H. Hammack C. Ogden S. C. Wen Z. Qian X. Li Y. (2016). Zika Virus Infects Human Cortical Neural Progenitors and Attenuates Their Growth. *Cell Stem Cell* 18 587–590. 10.1016/j.stem.2016.02.016 26952870 PMC5299540

[B50] Town T. Bai F. Wang T. Kaplan A. T. Qian F. Montgomery R. R. (2009). Toll-like receptor 7 mitigates lethal West Nile encephalitis via interleukin 23-dependent immune cell infiltration and homing. *Immunity* 30 242–253. 10.1016/j.immuni.2008.11.012 19200759 PMC2707901

[B51] van der Linden V. Pessoa A. Dobyns W. Barkovich A. J. Júnior H. V. Filho E. L. (2016). Description of 13 Infants Born During October 2015-January 2016 With Congenital Zika Virus Infection Without Microcephaly at Birth - Brazil. *MMWR. Morb. Mortal. Wkly. Rep.* 65 1343–1348. 10.15585/mmwr.mm6547e2 27906905

[B52] Venere M. Han Y. G. Bell R. Song J. S. Alvarez-Buylla A. Blelloch R. (2012). Sox1 marks an activated neural stem/progenitor cell in the hippocampus. *Development* 139 3938–3949. 10.1242/dev.081133 22992951 PMC3472585

[B53] Vermillion M. S. Lei J. Shabi Y. Baxter V. K. Crilly N. P. McLane M. (2017). Intrauterine Zika virus infection of pregnant immunocompetent mice models transplacental transmission and adverse perinatal outcomes. *Nat. Commun.* 8:14575. 10.1038/ncomms14575 28220786 PMC5321801

[B54] Vig P. J. Wei J. Shao Q. Lopez M. E. Halperin R. Gerber J. (2012). Suppression of calbindin-D28k expression exacerbates SCA1 phenotype in a disease mouse model. *Cerebellum* 11 718–732. 10.1007/s12311-011-0323-9 22076800

[B55] Waters A. M. Asfahani R. Carroll P. Bicknell L. Lescai F. Bright A. (2015). The kinetochore protein, CENPF, is mutated in human ciliopathy and microcephaly phenotypes. *J. Med. Genet.* 52 147–156. 10.1136/jmedgenet-2014-102691 25564561 PMC4345935

[B56] Wu K. Y. Li X. F. Zuo G. Ye Q. Deng Y. Q. Huang X. Y. (2016). Vertical transmission of Zika virus targeting the radial glial cells affects cortex development of offspring mice. *Cell Res.* 26 645–654. 10.1038/cr.2016.58 27174054 PMC4897185

[B57] Zhao H. Fernandez E. Dowd K. A. Speer S. D. Platt D. J. Gorman M. J. (2016). Structural basis of Zika Virus-specific antibody protection. *Cell* 166 1016–1027. 10.1016/j.cell.2016.07.020 27475895 PMC4983199

