# Supplementary material for: Congenital Zika Virus Infection in Immunocompetent Mice Causes Postnatal Growth Impediment and Neurobehavioral Deficits
Source: Front Microbiol. 2018 Aug 29;9:2028. doi: 10.3389/fmicb.2018.02028 (PMC6124374; doi:10.3389/fmicb.2018.02028)
Supplement: Supplementary file 1 [file Data_Sheet_1.pdf]

Supplemental Material

Congenital Zika virus infection in immunocompetent mice causes postnatal growth impediment and neurobehavioral deficits

Amber M. Paul, Dhiraj Acharya, Biswas Neupane, E. Ashley Thompson, Gabriel Gonzalez-Fernandez, Katherine M. Copeland, Me'Lanae Garrett, Haibei Liu, Mariper E. Lopez, Matthew de Cruz, Alex Flynt, Jun Liao, Yan-Lin Guo, Federico Gonzalez-Fernandez, Parminder J. S. Vig and Fengwei Bai

Materials and Methods

Quantitative PCR (qPCR) and plaque forming assay

Total RNA was extracted from tissues or cultured cells by using TRI Reagent and converted to cDNA using iSCRIPT cDNA synthesis kit (Bio-Rad). QPCR assays were performed using iTAQ polymerase supermix for probe-based assays (Bio-Rad) or iQ SYBR Green Supermix (Bio-Rad). Viral RNA of ZIKV *envelope (E)* [1] was measured by qPCR and infectious viruses were measured by standard plaque assay. Threshold cycle values that were $\geq$ 39 cycles were excluded from the qPCR results, and 1 PFU per volume of sample was set as the limit of viral detection for the plaque assays. Murine primers were designed according to previous publications and are listed in supplemental table 1.

Neurobehavioral Test.

Cliff Recognition Test. Mice were placed on a platform (2-inch wide, 11-inch long, and 1-inch high) that divided the center of a clear plexiglass box. One side of the box was placed on a table with a checkered pattern below "platform side", while the other half hung over the edge of the table to simulate a "fall side". Mice were measured from the time the mice were placed on the center platform to the side chosen (seconds) and the type of side chosen, i.e. fall side, platform side or being static (no side chosen) [2,3]. The test was blinded from the investigator.

Eye histology. Right eyes and orbits from ZIKV- and mock-pups were exenterated and immediately placed in 10% formalin for 72 h at 4°C. Excess orbital soft tissue was dissected free of the globe and the eyelids and the remaining tissue was embedded in paraffin. The globes were serially sectioned at 5 μm in the sagittal orientation. Sections going through the optic nerve and pupil were stained with haematoxylin & eosin (Leica), and imaged by bright-field microscopy.

Immunohistochemistry and immunofluorescence. Midsagittal, coronal brain sections and cervical spinal cords (6 μm) of D19 and D40 p.b. mce were probed with Calbindin-D28k, Caspase-9, NeuN, and GFAP specific antibodies, followed by labeling with Alexa 488 or 546 secondary antibodies (Invitrogen) and DAPI for immunofluorescence, or biotinylated secondary antibody, developed with ExtrAvidin peroxidase immunostaining kit (Sigma), for immunohistochemistry. The slides were mounted with Aqua-mount (Fisher Scientific) and observed using an Epi-fluorescence (Olympus BX60) or bright-field microscope, and images were captured using a digital camera (DP70).

Statistical analyses. Data were compared with either a two-tailed Student's *t*-test, a Mann-Whitney U test, or a Fisher's exact test by using GraphPad Prism software (version 6.0), and $p < 0.05$ was considered statistically significant.

Supplemental Figure Legends

Figure S1. ZIKV-infection induces antiviral cytokine and humoral responses, but does not alter body weight in dams. Dams were infected with ZIKV (10^4 PFU, i.p.) or mock infected on E8.5, and blood samples were collected on D1 and D21 p.i.. (**A**) Viral RNA loads in dam's blood (n = 4-6 dams). **(B)** Plaque reduction neutralization testing (PRNT) of D21 plasma samples against ZIKV (n = 2 dams). **(C)** Cytokine gene expression in blood of ZIKV-infected dams on D1 p.i. (n =5-6 dams), horizontal line represents mock controls set at 1. **(D)** Weight of dams during pregnancy (E8.5), and after delivery on D0, D12 and D19 (n = 5-9 dams).

Figure S2. ZIKV-pups displayed trends in balance and decision-making disparities, without displaying ocular tissue disparities. Pups born to dams infected with ZIKV (10^4 PFU, i.p.), or mock infected on E8.5 were subjected to the cliff recognition test of balance and decision making. Pups were placed on a center bar; percentage of pups that chose the platform side (green), fall side (red), or no side chosen (static, blue) out of total pups tested was measured per group on D15, 19, 40 and 60 p.b., (n = 11-38 pups). Data were analyzed using a Fisher's exact test (C, $p > 0.05$). Center values are means. (**B**) Anterior ocular structures including the cornea, iris and lens showed normal histology and anatomic relationships, and were free of inflammation at D40 p.b.. The corneal

epithelium (asterisk) and endothelium (arrow) were within normal limits, top left panel. Eyelids and dermal adnexal structures showed normal architecture relationships at D40 p.b. with the palpebral conjunctiva containing scattered goblet cells (arrow, top right panel). Asterisk identifies the bulbar conjunctiva. Angle (arrow) and ciliary body at D40 p.b. were well formed and showed normal histological features, bottom left panel. Full thickness cross-section of the mid-peripheral retina at D19 p.b. The retina showed normal lamination and cytological differentiation of the neuronal layers, photoreceptors, retinal-pigmented epithelium (arrow), and choroid without degenerative changes, atrophy or inflammation, bottom right panel. Abbreviations: GC, ganglion cells; IPL, inner plexiform layer; INL, inner nuclear layer; OPL, outer plexiform layer; ONL, outer nuclear layer; IS, inner segments; OS, outer segments. The sections were stained with haematoxylin and esosin, and photographed at 200× and 400× original magnifications in the top and bottom panels, respectively.

Figure S3. Increased apoptosis and dysregulated cortical layer 1 neuronal migration in ZIKV-pups brains is coupled with increased astrogliosis in cervical spinal cords. Dams were infected with ZIKV (1×10^4 PFU, i.p.) or mock infected on E8.5. **(A)** Confocal micrographs of Caspase 9 (Red), NeuN (green) and DAPI (blue) labeled cortical sections of pup's brains at D19 p.b., scale bar = 20 μm. Images represent three biological samples. **(B)** Mean fluorescent intensity (MFI) of Caspase-9 / DAPI and Caspase-9 / NeuN were measured using ImageJ software. (**C**) Calbindin-D28k positive (green) neuronal cells in the brains of D19 pups migrating into layer 1 (1000× original magnification). **(D)** Confocal images of the cervical spinal cords were probed with GFAP (brown) and haematoxylin and eosin (purple). Arrows denote motor neurons, and arrow heads denote

GFAP-labeled astrocytes. 400× original magnification. Data were compared to the control by using a Mann-Whitney U test (* denotes $p < 0.05$; center values are means and error bars represent s.e.m.). Images represent three biological samples from each group.

References

1 Acharya, D. *et al.* An ultrasensitive electrogenerated chemiluminescence-based immunoassay for specific detection of Zika virus. *Sci Rep* **6**, 32227, doi:10.1038/srep32227 (2016).

2 Fox, M. W. The visual cliff test for the study of visual depth perception in the mouse. *Anim Behav* **13**, 232-233 (1965).

3 Godar, S. C. *et al.* Maladaptive defensive behaviours in monoamine oxidase A-deficient mice. *Int J Neuropsychopharmacol* **14**, 1195-1207, doi:10.1017/S1461145710001483 (2011).

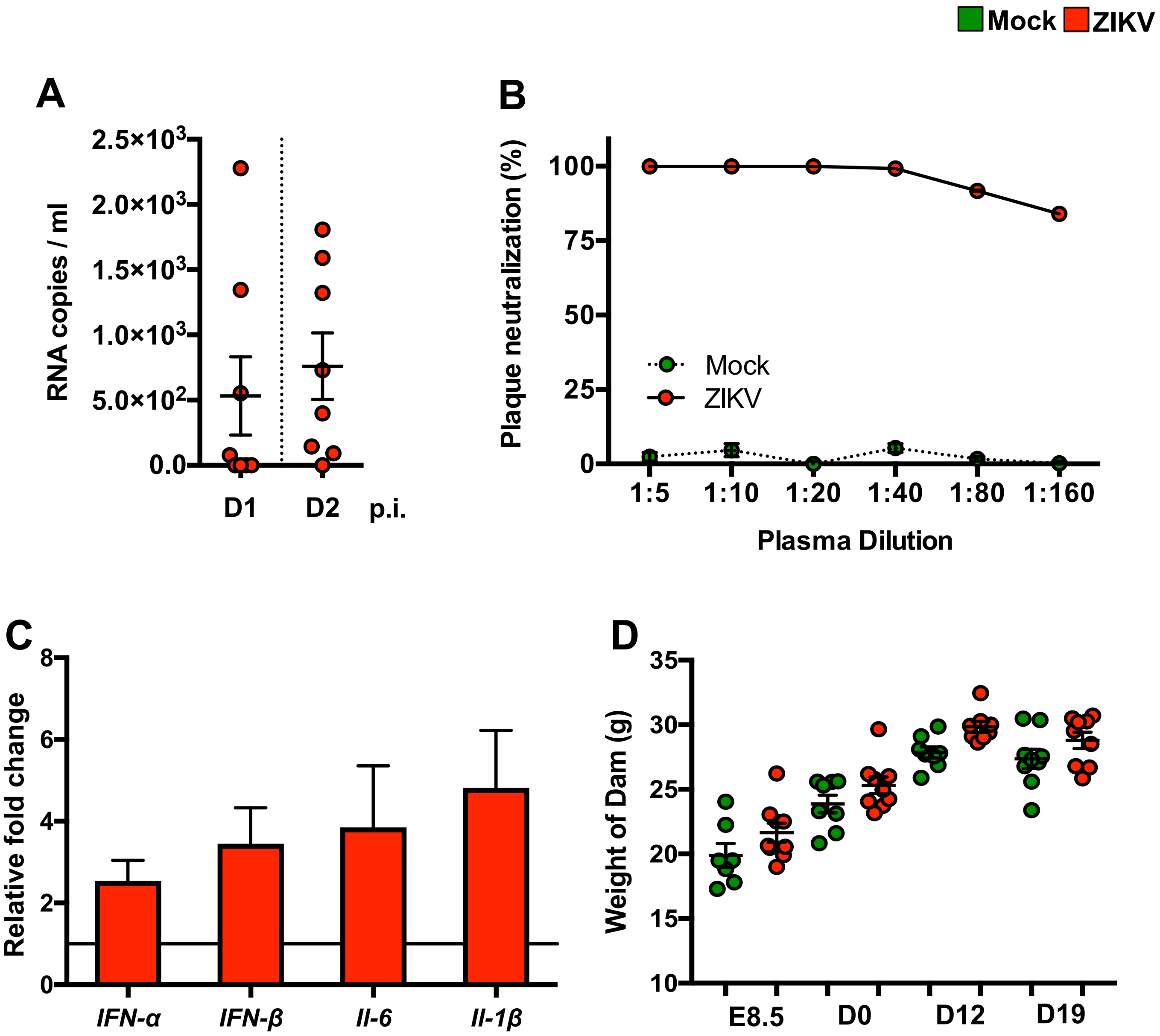
Mock
ZIKV
A
RNA copies / ml
2.5×10^3
2.0×10^3
1.5×10^3
1.0×10^3
5.0×10^2
0.0
D1
D2
p.i.
B
Plaque neutralization (%)
100
75
50
25
0
Mock
ZIKV
1:5
1:10
1:20
1:40
1:80
1:160
Plasma Dilution
C
Relative fold change
8
6
4
2
0
IFN-α
IFN-β
Il-6
Il-1β
D
Weight of Dam (g)
35
30
25
20
15
10
E8.5
D0
D12
D19

Fig. S1

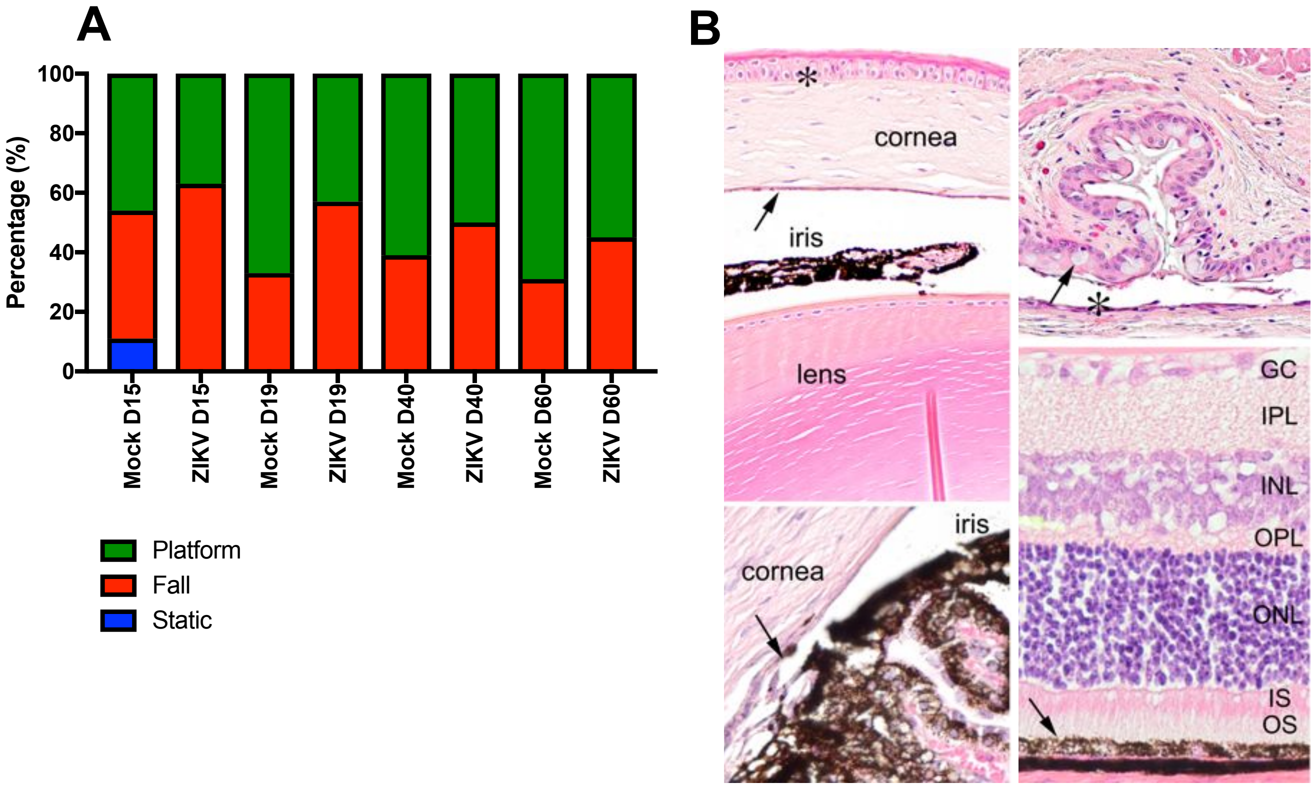
A
100
80
60
40
20
0
Percentage (%)
Mock D15
ZIKV D15
Mock D19
ZIKV D19
Mock D40
ZIKV D40
Mock D60
ZIKV D60
Platform
Fall
Static
B
cornea
iris
lens
cornea
iris
GC
IPL
INL
OPL
ONL
IS
OS

Fig. S2

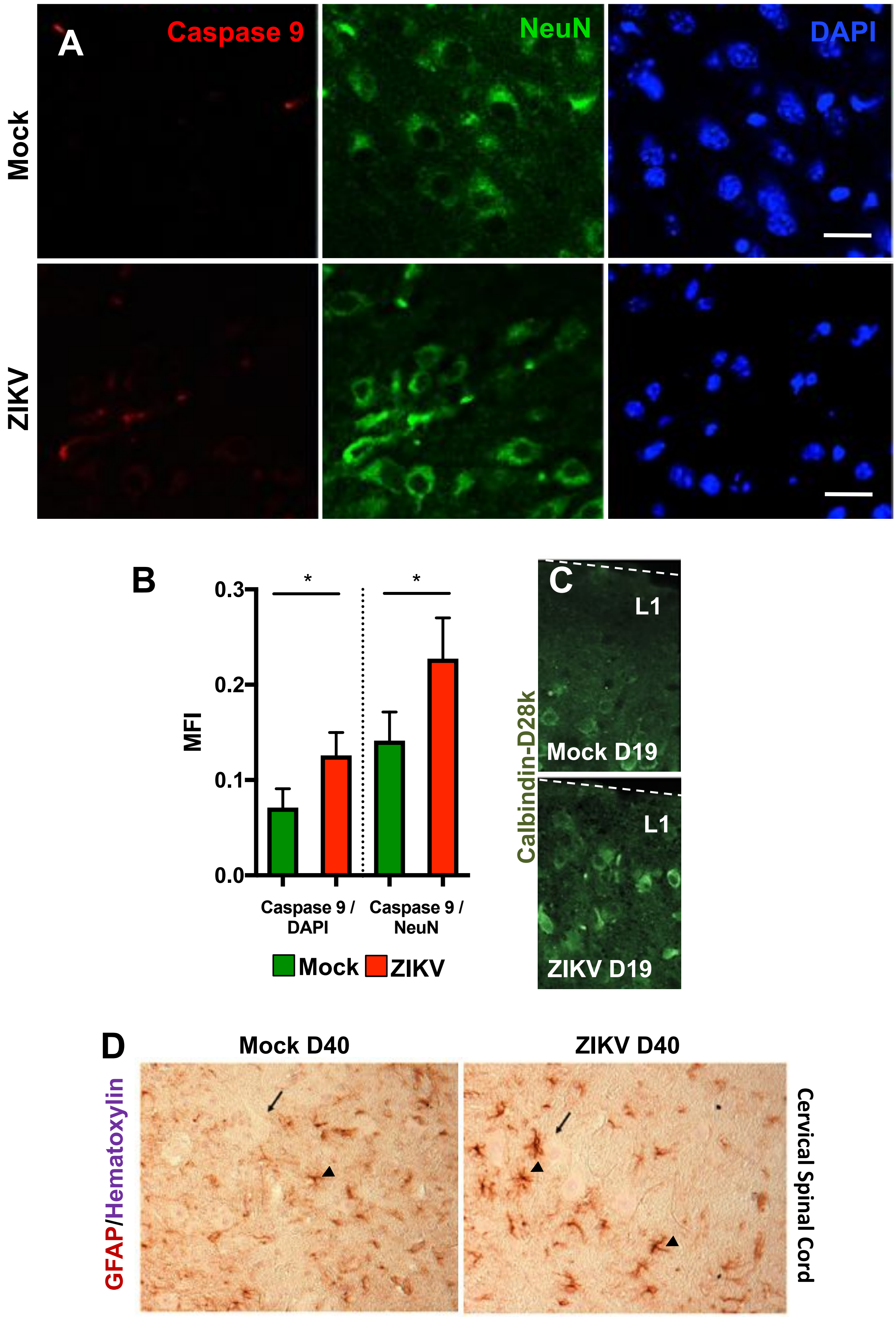

A
Caspase 9
NeuN
DAPI
Mock
ZIKV
B
0.3
0.2
0.1
0.0
MFI
*
*
Caspase 9 / DAPI
Caspase 9 / NeuN
Mock
ZIKV
C
L1
Calbindin-D28k
Mock D19
L1
ZIKV D19
D
Mock D40
ZIKV D40
GFAP/Hematoxylin
Cervical Spinal Cord

Fig. S3

Supplemental Table 1.

Primer	Sense (5'-3')	Antisense (5'-3')
β-Actin[1]	AGA GGG AAA TCG TGC GTG AC	CAA TAG TGA TGA TGA CCT GGC CGT
Sox-1	CCC ATG CGC TAC GAC AT	CCG TAG CCC GAA GC GA
Cenpf[2]	CTT ACC CAG GAG TTA CAG CAA G	TGC CTG AAG AGC TTG TTC TG
Tbr2[2]	CCA CTG GAT GAG GCA GGA GAT T	GTC CTC TGT CAC TTC CAC GAT G
Ng2[3]	GGG CTG TGC TGT CTG TTG A	TGA TTC CCT TCA GGT AAG GCA
Nestin[3]	GCT GGA ACA GAG ATT GGA AGG	CCA GGA TCT GAG CGA TCT GAC
β-Tubulin III[3]	TGG ACA GTG TTC GGT CTG G	CCT CCG TAT AGT GCC CTT TGG
Mmp15	ATG GCA CCC TTC TAC CAG TG	ACC ATC TGG GGA GCC ATA CA
Ifnα	AGG ACA GGA AGG ATT TTG GA	GCT GCT GAT GGA GGT CAT T
Ifnβ	CGT TCC TGC TGT GCT TCT CC	TCT TGG AGC TGG AGC TGC TT
Il-6[4]	CGG CCT TCC CTA CTT CAC AA	TCC ACG ATT TCC CAG AGA ACA
Il-1β[5]	TGG TGT GTG ACG TTC CCA TT	CAG CAC GAG GCT TTT TTG TTG

Supplemental Table References

1. F. Bai *et al.*, Use of RNA interference to prevent lethal murine west nile virus infection. *The Journal of infectious diseases* **191**, 1148 (2005).
2. C. Li *et al.*, Zika Virus Disrupts Neural Progenitor Development and Leads to Microcephaly in Mice. *Cell stem cell* **19**, 120 (2016).
3. Y. L. Guo, J. Ye, F. Huang, p38alpha MAP kinase-deficient mouse embryonic stem cells can differentiate to endothelial cells, smooth muscle cells, and neurons. *Developmental dynamics : an official publication of the American Association of Anatomists* **236**, 3383 (2007).
4. D. Lai, F. Wang, Z. Dong, Q. Zhang, Skin-derived mesenchymal stem cells help restore function to ovaries in a premature ovarian failure mouse model. *PloS one* **9**, e98749 (2014).
5. K. Isoda *et al.*, Deficiency of interleukin-1 receptor antagonist promotes spontaneous femoral artery aneurysm formation in mice. *The American journal of pathology* **180**, 1254 (2012).
